# Impact of Missense Mutations on Spike Protein Stability and Binding Affinity in the Omicron Variant

**DOI:** 10.3390/v16071150

**Published:** 2024-07-17

**Authors:** Vidhyanand Mahase, Adebiyi Sobitan, Qiaobin Yao, Xinghua Shi, Hong Qin, Dawit Kidane, Qiyi Tang, Shaolei Teng

**Affiliations:** 1 Department of Biology, Howard University, Washington, DC 20059, USA; 2 Department of Computer & Information Sciences, Temple University, Philadelphia, PA 19122, USA; 3 Department of Computer Science and Engineering, University of Tennessee at Chattanooga, Chattanooga, TN 37403, USA; 4 Department of Physiology and Biophysics, Howard University College of Medicine, Washington, DC 20059, USA; 5 Department of Microbiology, Howard University College of Medicine, Washington, DC 20059, USA

**Keywords:** SARS-CoV-2, COVID-19, Spike protein, Omicron Variant, computational saturation mutagenesis

## Abstract

The global effort to combat the COVID-19 pandemic faces ongoing uncertainty with the emergence of Variants of Concern featuring numerous mutations on the Spike (S) protein. In particular, the Omicron Variant is distinguished by 32 mutations, including 10 within its receptor-binding domain (RBD). These mutations significantly impact viral infectivity and the efficacy of vaccines and antibodies currently in use for therapeutic purposes. In our study, we employed structure-based computational saturation mutagenesis approaches to predict the effects of Omicron missense mutations on RBD stability and binding affinity, comparing them to the original Wuhan-Hu-1 strain. Our results predict that mutations such as G431W and P507W induce the most substantial destabilizations in the Wuhan-Hu-1-S/Omicron-S RBD. Notably, we postulate that mutations in the Omicron-S exhibit a higher percentage of enhancing binding affinity compared to Wuhan-S. We found that the mutations at residue positions G447, Y449, F456, F486, and S496 led to significant changes in binding affinity. In summary, our findings may shed light on the widespread prevalence of Omicron mutations in human populations. The Omicron mutations that potentially enhance their affinity for human receptors may facilitate increased viral binding and internalization in infected cells, thereby enhancing infectivity. This informs the development of new neutralizing antibodies capable of targeting Omicron’s immune-evading mutations, potentially aiding in the ongoing battle against the COVID-19 pandemic.

## 1. Background

In April 2022, the Omicron subvariants, specifically BA.4/BA.5, were identified and rapidly propagated around the globe. During early July 2022, these variants had grown to constitute nearly 80% of all COVID-19 cases in America [1]. As the virus undergoes continuous evolution and accumulates genetic changes, the Omicron variant has been classified as a Variant of Concern (VOC), contributing to a significant upsurge in new cases, surpassing 400,000 cases in the United States alone [2]. VOCs pose significant challenges due to their demonstrated increased transmissibility, severe disease manifestations, enhanced ability to evade diagnostic tests, and reduced neutralization by antibodies from vaccinations. The Omicron variant has outpaced other VOCs, bearing a higher number of mutations, particularly in the Spike (S) protein [3]. Given the multitude of S protein mutations, it is possible that many of these missense mutations impact the S protein’s stability and binding affinity to its human receptors. The computational saturation mutagenesis offers a rapid approach to predict the functional implications of mutations on protein stability and protein–protein interactions of coronavirus S proteins [4,5,6].

The S glycoprotein is the crucial protein that determines viral host selection and pathology, making it a target for both diagnostic and therapeutic interventions. The S_1_ domain, responsible for receptor binding, is divided into an N-terminal domain (NTD: 14–305) and a receptor-binding domain (RBD: 319–541) [7]. Notably, the receptor-binding motif (RBM: 437–508) of the RBD directly interacts with the ACE2 receptor, highlighting the significance of this region in the infection process. A recent study identified 22 amino acid substitutions in the RBM of the Omicron variant, with specific positions (475–477, 489, 493, and 501) attributed to its heightened binding ability to the receptor [8]. The closely related BA.4 and BA.5 variants share lineage with the Omicron BA.2 variant, but exhibit unique mutations, including A484E, R498Q, and Y501N, in their amino acid sequences compared to their predecessor [9]. Notably, the Omicron variant surpasses previous variants with the highest number of mutations in the RBM, such as A484E and R493Q, associated with immune evasion by reducing bindings to host antibodies [10]. To gain a deeper understanding of the Omicron variant’s impact on infection rates and its ability to evade immunity, it is crucial to unravel the molecular mechanisms governing its receptor-recognition capabilities [11].

SARS-CoV-2, in contrast to past seasonal coronaviruses and influenza A, demonstrates a markedly accelerated rate of evolution and propagation. Significantly, many monoclonal antibodies targeting the RBM have shown reduced efficacy in neutralizing the Omicron variant, although some antibodies continue to recognize Omicron through antigenic binding sites outside the RBM [12]. Analyzing the atomic-level structure of SARS-CoV-2 and its variants provides valuable insights into their interactions with susceptible cells during infection. This knowledge aids in identifying robust target regions for neutralizing antibodies, supporting the development of drugs and vaccines to combat SARS-CoV-2. In this study, we applied structure-based computational strategies to investigate all S mutations in Omicron and Wuhan-Hu-1 that affect protein stability (ΔΔG). Our results predict how the S RBM mutations might affect the binding affinity (ΔΔΔG) within both Omicron-S–ACE2 and Wuhan-Hu-1-S–ACE2 complexes. Our findings shed light on specific residues that warrant further experimental validation, which is essential for designing effective neutralizing peptides to address the potential immunogenicity of the SARS-CoV-2 Omicron Variant.

## 2. Materials and Methods

### 2.1. Structure Collection

To investigate the impacts of S RBD mutations on the protein stability and binding, we compared the Omicron-S (PDB ID: 7WBP)—a crystal structure of the RBD of Omicron variant S in complex with its receptor human ACE2—with the original strain Wuhan-Hu-1 S RBD–ACE 2 complex (PDB ID: 6LZG). Both structures were obtained from the Protein Data Bank [13]. Structural alignments and 3D structural images were generated using PyMol’s ‘fetch’ and ‘align’ commands [14].

### 2.2. Free Energy Calculations

The FoldX [15] was employed to calculate the free energies, investigating the effects of RBD mutations on protein stability and binding affinity in both the Omicron S–ACE2 and Wuhan-Hu-1 S–ACE2 complexes. Prior to energy calculations, all protein structures were repaired using the ‘RepairPDB’ command. The FoldX was utilized to perform computational saturation mutagenesis, total energy calculations. van der Waals interactions, hydrogen bonding, etc. To analyze the effects of mutations on proteins, a Perl script was executed to generate lists of systematic changes in each residue. These lists were used within FoldX software (2023) to model the impact of mutations on the S RBD protein and the RBD–ACE2 complex.

The change in free folding energy (ΔΔG) representing the difference between the mutant and wild-type protein free energy was computed using the repaired structures. The “BuildModel” function of FoldX generated mutant models for each desired protein by calculating the free energy change (ΔG) between a wildtype structure and its mutated version. This difference in folding free energy (ΔΔG), where negative values indicate increased stability and positive values denote destabilization, was used to assess the impact of mutations on the overall protein conformation. Computational saturation mutagenesis was performed on the S-RBD of both Wuhan-Hu-1 and Omicron strains. For free-folding calculations, the stability values between the mutant (MUT) and wild-type (WT) residues were computed as follows:

ΔG _(folding)_ = G _(folded)_ − G _(unfolded)_ (1)

ΔΔG _(stability)_ = ΔG _(folding)_ MUT − ΔG _(folding)_ WT (2)

The ‘AnalyseComplex’ command calculated the free-folding energy change between the interactions (binding energy change). By disassembling each protein and evaluating their distinct energies, the ΔΔG _(binding)_ was determined. This value was then subtracted from the energy of the entire protein complex to derive the change in binding-free energy (ΔΔΔG) by subtracting it from the wild-type complex. The change in binding-free energy (ΔΔΔG) between the mutant and wild-type structures is given by the following equations:

ΔΔG _(binding)_ = ΔG _(folding: AB)_ − ΔG _(folding: A)_ − ΔG _(folding: B)_ (3)

ΔΔΔG _(binding)_ = ΔΔG _(binding)_MUT − ΔΔG _(binding)_WT (4)

The folding energy changes (ΔΔG) were classified into the following five groups: large decrease in stability (ΔΔG > 2.5 kcal/mol), moderate decrease (0.5 < ΔΔG ≤ 2.5 kcal/mol), neutral (−0.5 < ΔΔG ≤ 0.5 kcal/mol), moderate increase (−0.5 ≤ ΔΔG < −2.5 kcal/mol), and large increase (ΔΔG < −2.5 kcal/mol). In the energy calculations, binding energy values are often smaller in magnitude than stability values due to stronger interactions formed during binding and favorable enthalpic contributions outweighing entropic losses. Additionally, computational methods differ, with binding energy calculations focusing on the protein interface and stability calculations considering the entire protein, leading to variations in energy magnitudes. The binding energy changes (ΔΔΔG) were categorized into the following five classifications: large decrease in binding affinity (ΔΔΔG > 0.5 kcal/mol), moderate decrease (0.1 < ΔΔΔG ≤ 0.5 kcal/mol), neutral (−0.1 < ΔΔΔG ≤ 0.1 kcal/mol), moderate increase (−0.5 ≤ ΔΔΔG < −0.1 kcal/mol), and large increase (ΔΔΔG < −0.5 kcal/mol). The R package was used to generate heatmaps and line graphs for ΔΔG and ΔΔΔG values [16].

### 2.3. Sequence-Based Analysis

For sequence analysis, the S RBD amino acid sequences of Wuhan-Hu-1-S–ACE2 and Omicron-S–ACE2 were extracted from PDB (PDB ID: 6LZG and 7WBP). The full-length S sequence was obtained from Uniprot (ID: P0DTC2) [17]. A pairwise sequence alignment with an EMBOSS Needle (https://www.ebi.ac.uk/jdispatcher/psa/emboss_needle, accessed on 1 March 2024) was used to align the FASTA sequences of the Wuhan and Omicron spike proteins [18].

### 2.4. Mutation Collection

Omicron viral mutations were collected from the Stanford University Coronavirus Antiviral and Resistance Database (https://covdb.stanford.edu/) [19] and The National Genomics Data Center (NGDC), part of the China National Center for Bioinformation (CNCB) (https://ngdc.cncb.ac.cn/ncov/variation/annotation, accessed on 1 March 2024) [20].

## 3. Results

### 3.1. Effects of Omicron Mutations on Protein Stability

**Figure 1:**
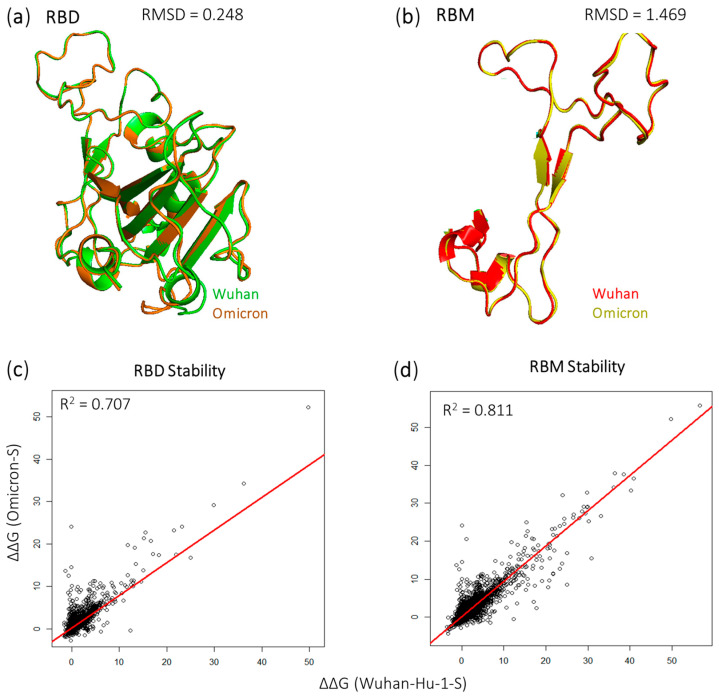

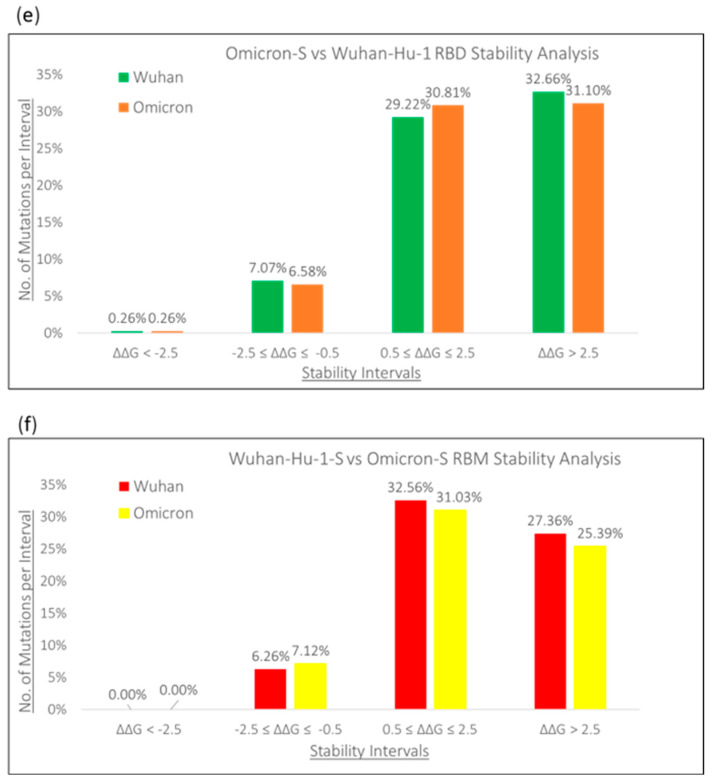
Structural alignment displaying the (**a**) RBD and (**b**) RBM of Wuhan-S/ Omicron-S proteins. The regression analysis shows ΔΔG values of RBD (**c**) and RBM (**d**) mutations of Wuhan-S/ Omicron-S proteins. Bar charts depictinWuhan-S/Omicron-S proteins distribution effects of mutations affecting RBD (**e**) and RBM (**f**) stability.

We performed structural alignment on the Wuhan-Hu-1-S and Omicron-S proteins in the Receptor-Binding Domain (RBD) and Receptor-Binding Motif (RBM) regions (Figure 1a,b). The root-mean-square deviation (RMSD) value indicates a high degree of similarity for the RBD = 0.248 over 195 residues, while the similarity is acceptable in the RBM with an RMSD = 1.469 over 72 residues. Our analysis extended to evaluating the folding energy changes (ΔΔG) associated with all potential RBD and RBM mutations, aiming to estimate their impact on protein stability. Notably, the correlation analysis of ΔΔG demonstrated an r^2^ value of 0.707 in RBD (Figure 1c), and 0.811 in the RBM (Figure 1d). This indicates that mutations in the RBM region may have a more consistent impact on protein stability compared to the RBD.

The folding energy changes (ΔΔG) were classified as highly stabilizing (ΔΔG < −2.5 kcal/mol), moderately stabilizing (−2.5 ≤ ΔΔG < −0.5 kcal/mol), moderately destabilizing (0.5 < ΔΔG ≤ 2.5 kcal/mol), and highly destabilizing (ΔΔG > 2.5 kcal/mol). In the RBD, our analysis involved 3861 mutations for Wuhan-Hu-1-S and 3934 mutations for Omicron-S (Figure 1e). For highly stabilizing mutations, both S proteins were similar (0.26%). The Wuhan-Hu-1-S was greater in the moderately stabilizing (7.07%) and the highly destabilizing intervals (32.66%). However, the Omicron-S (30.81%) was greater in the moderately stabilizing category than the Wuhan-Hu-1-S (29.22%). Moving to the RBM, we analyzed 1417 mutations from the Omicron-S and 1422 mutations from the Wuhan-S. No highly stabilizing mutations were reported in the RBM for both complexes. As shown in Figure 1f, the Omicron-S displayed a more moderate stabilizing effect (7.12%) versus the Wuhan-S (6.26%). Conversely, the Wuhan-Hu-1-S displayed a higher percentage in both moderately destabilizing (32.56%) and highly destabilizing (27.36%) mutations.

### 3.2. Key Residues Predicted to Affect the RBD Stability of Omicron

**Figure 2:**
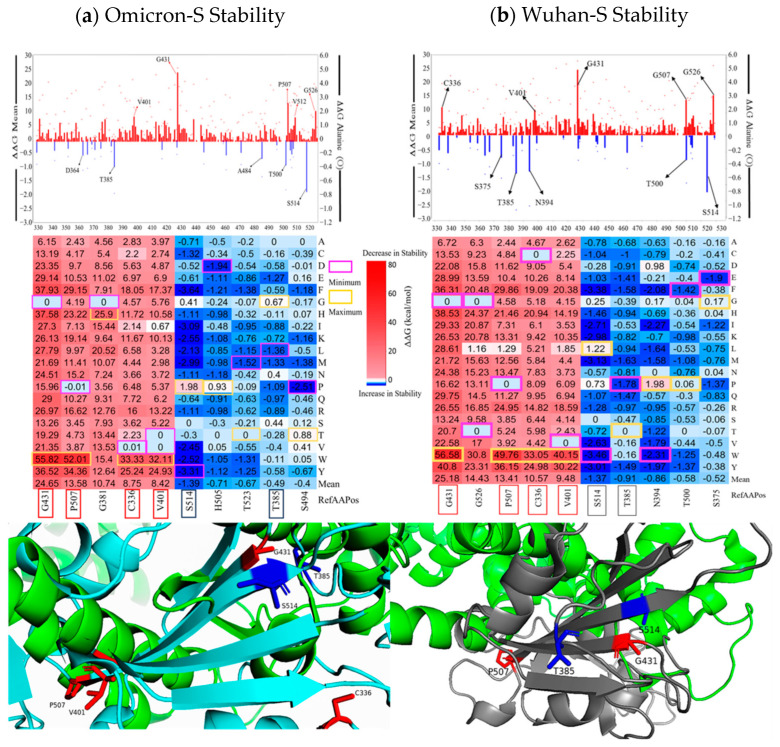
Line graphs and heatmaps displaying (**a**) Omicron and (**b**) Wuhan-Hu-1 S RBD top destabilizing/stabilizing values. Omicron-S is displayed in cyan, while the Wuhan-Hu-1-S is gray. ACE2 is depicted as green. The red squares indicate common destabilizing residues and positions in both heatmaps, while the blue squares indicate common stabilizing residues and positions in both heatmaps. The minimum energy values are in magenta, and the maximum energy values are in yellow.

In Figure 2, we illustrated line charts displaying the mean ΔΔG values for each residue position of the spike RBD proteins, as well as the corresponding ΔΔG values for alanine substitutions. Within the Omicron S-protein, the mutations in residue G431 showed the most significant destabilizing effects (mean ΔΔG = 24.65 kcal/mol), whereas the mutations in S514 exhibited the highest stabilizing effects (mean ΔΔG = −1.39 kcal/mol). A similar trend was observed in the Wuhan S-protein, with ΔΔG values ranging from 25.18 kcal/mol at G431 to −1.37 kcal/mol at S514. Heatmaps highlighting the residues with the greatest destabilizing and stabilizing effects on RBD protein stability, along with structural representations of these key residues, are also presented in Figure 2.

Our study predicts that G431W and P507W are the most destabilizing mutations common to both RBD proteins. The G431W mutation resulted in substantial decreases in stability for both Omicron S and Wuhan-Hu-1 S proteins (56.58 kcal/mol and 55.82 kcal/mol, respectively). This destabilization is due to the substitution of glycine (G), which disrupts the heterotrimers, impairs helix formation, and reduces the molecule’s overall stability. Moreover, it significantly diminishes interactions between extracellular molecules [21]. The P507W mutation (52.01 kcal/mol for Omicron-S and 49.76 kcal/mol for Wuhan-Hu-1) is particularly destabilizing when proline (P) is located internally in α-helices or β-sheets [22]. In contrast, tryptophan (W) promotes structural hydrophobic interactions among proteins, peptides, and biomolecules [23]. The presence of tryptophan-R groups within transmembrane domains is essential for maintaining the structural integrity of membrane-bound proteins.

### 3.3. Effects of Omicron-S and Wuhan-Hu-1-S Mutations on Binding Affinity

**Figure 3:**
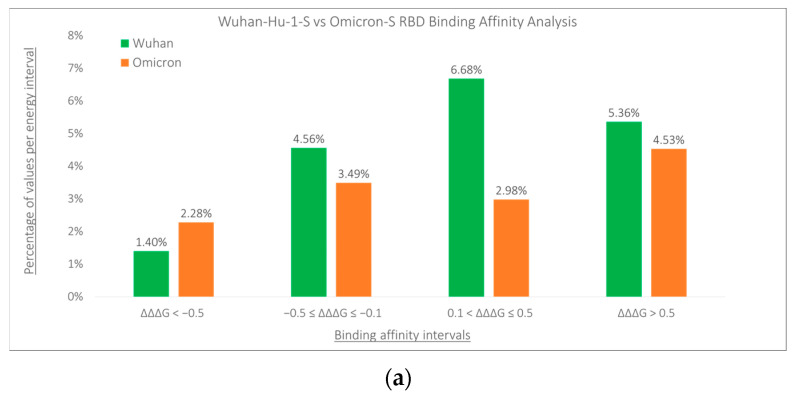

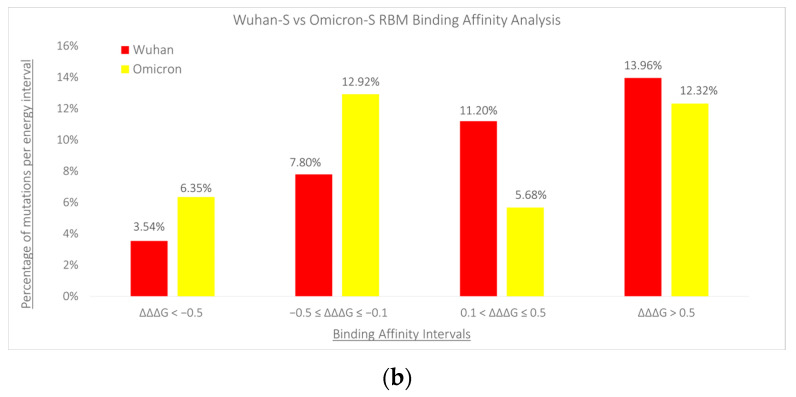
Bar charts displaying Wuhan-Hu-1-S vs. Omicron-S binding affinity values (ΔΔΔG) per energy interval in the RBD (**a**) and RBM (**b**) regions.

We investigated the potential differences in the effects of mutations on protein binding affinity within the binding regions of Wuhan-Hu-1-S and Omicron-S proteins. Our analysis involved assessing the binding energy changes (ΔΔΔG) of mutations and grouping the percentages of mutations based on specific intervals of ΔΔΔG values (Figure 3). In the RBD region, we evaluated 3860 mutations for Wuhan-Hu-1-S and 3727 mutations for Omicron-S. As shown in Figure 3a, Omicron-S showed a higher percentage (2.28%) compared to Wuhan-S (1.40%) (*p*-value = 1.26 × 10^−5^) for mutations displaying a high increase in binding affinity (ΔΔΔG < −0.5 kcal/mol). However, for moderate increases in binding affinity (−0.5 ≤ ΔΔΔG ≤ −0.1 kcal/mol), Omicron-S exhibited a slightly lower percentage (3.49%) compared to Wuhan-S (4.56%) (*p*-value = 0.466). Regarding mutations with moderate decreases in binding affinity (0.1 < ΔΔΔG ≤ 0.5 kcal/mol), Omicron-S also showed a lower percentage (2.98%) compared to Wuhan-S (6.68%) (*p*-value = 1.02 × 10^−29^). The impact of Omicron-S on a significant increase in binding affinity (ΔΔΔG > 0.5 kcal/mol) was also lower at 4.53% compared to 5.36% in Wuhan-S. In the RBM region, we analyzed 1411 mutations for Wuhan-Hu-1-S and 1355 mutations for Omicron-S. Interestingly, we observed that mutations in the Omicron-S displayed a higher percentage in stabilizing effects for protein complexes compared to Wuhan-S (Figure 3b). Notably, 6.35% of Omicron-S RBM mutations exhibited a significant increase in binding affinity (ΔΔΔG < −0.5 kcal/mol), surpassing the 3.54% observed in Wuhan-Hu-1-S RBM mutations (*p*-value = 6.867 × 10^−6^). Additionally, 12.92% of Omicron-S mutations displayed a moderate increase in binding affinity (−0.5 ≤ ΔΔΔG ≤ −0.1 kcal/mol), compared to 7.80% of Wuhan-S mutations (*p*-value = 1.24 × 10^−5^). Conversely, Omicron-S showed a lower percentage (5.68%) compared to Wuhan-S (11.20%) for mutations exhibiting a moderate decrease in binding affinity. Furthermore, the Omicron-S variant showed a lower impact on a significant decrease in binding affinity (ΔΔΔG > 0.5 kcal/mol), with a rate of 12.32% compared to 13.96% in the Wuhan-Hu-1-S variant.

### 3.4. Key Sites Predicted to Alter the Omicron S RBD–ACE2 Binding Affinity

**Figure 4:**
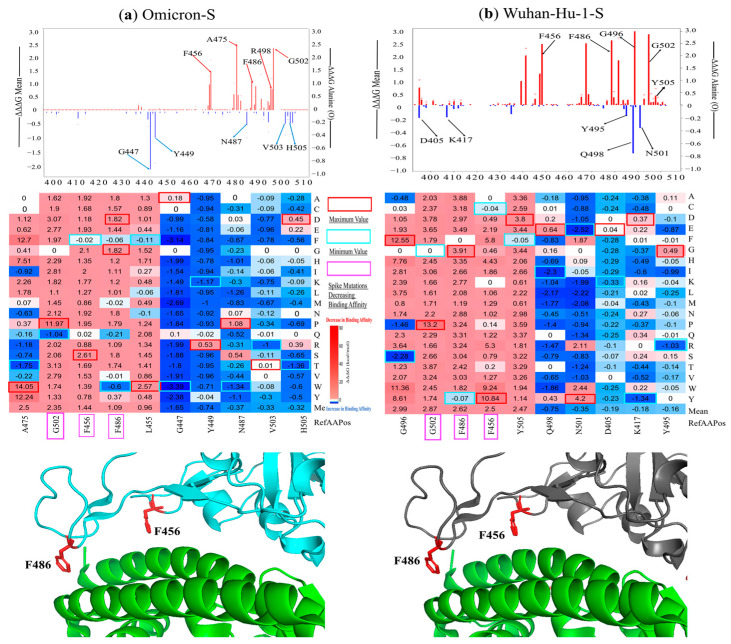
Key Spike positions and residues in ACE2-Omicron-S (**a**) and ACE2-Wuhan-HU-1-S (**b**) complexes. ACE2 is shown in green, the Wuhan-Hu-1-S is gray, and the Omicron-S is cyan. PyMol visuals show mutations causing an increase in binding affinity, which are shown in red. The heatmaps of key spike mutations, maximum values (red), minimum (cyan), and mutations decreasing binding affinity (fuchsia) are also indicated.

The mean ΔΔΔG values for each residue position of the spike RBD and RBM regions are shown in Figure 4. Notably, mutations at residues G502, F486, and F456 significantly reduce the binding affinity of both Omicron-S and Wuhan-Hu-1-S complexes. The mutations in G502 exhibit the most destabilizing effects for the Omicron S RBD–ACE2 complex (mean ΔΔΔG = 2.35 kcal/mol). Particularly noteworthy is the G502P mutation, which exerts the maximum destabilizing effect (ΔΔΔG = 11.97 kcal/mol) among all mutations analyzed.

### 3.5. Predicted Changes in Binding Affinity of Omicron-S and Wuhan-Hu-1-S

**Figure 5:**
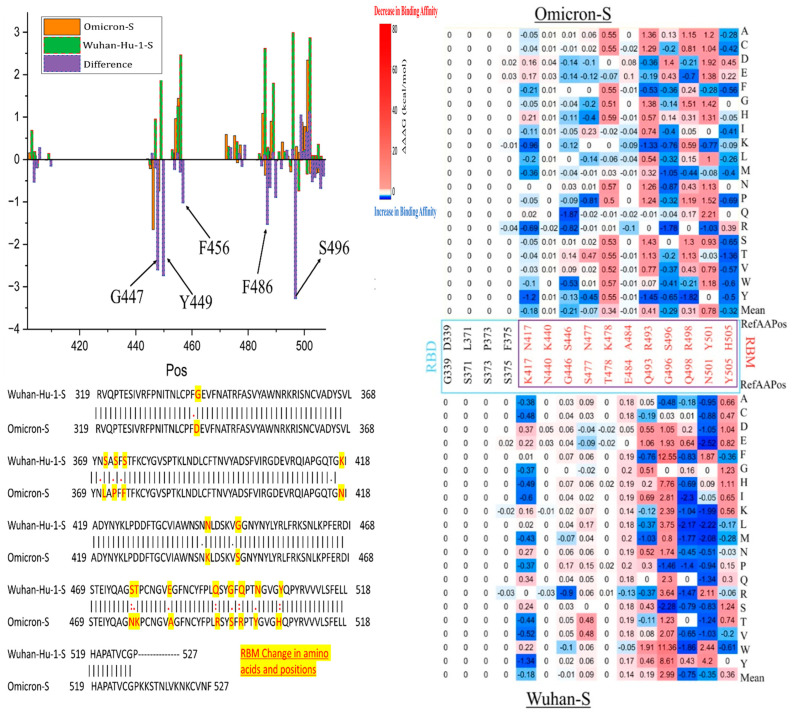
Changes in binding affinity of Omicron-S–ACE2 and Wuhan-Hu-1-S–ACE2 complexes.

We observed the distinct effects of mutations on predicted binding affinity in certain positions of Omicron-S compared to the Wuhan-Hu-1-S (Figure 5). Notably, some mutations at certain positions of Omicron-S, including Y449, G447, and 496, can stabilize the Omicron-S RBD–ACE2 complex, but destabilized the Wuhan-Hu-1-S RBD–ACE2 complex. Mutations in residues F456 and F486 significantly reduce the binding affinity of the Wuhan-Hu-1-S RBD–ACE2 complex. Located in the ACE2–RBD interface, F456 and F486 interact with ACE2 residues 19 to 42, altering the electrostatic surface charges at the interface. This process can also affect antibody–drug binding due to the larger size of the mutated side chains [24].

Specifically, the F456A (ΔΔΔG mean = 3.36 kcal/mol in Wuhan-Hu-1-S, and 1.92 kcal/mol in Omicron-S) is a class 1 epitope that generally weakened immunization levels by vaccine sera, though it has a limited effect on convalescent sera [25]. Moreover, this mutation is not found in native sequences, and significantly decreases viral entry titers. The F456 of the Omicron variant formed a strong hydrogen bond with Q24 and T27 found in ACE2, whereas this bond is lacking in other VOCs [11]. Another example is the F486V mutation, which has a ΔΔΔG of 3.03 kcal/mol in Wuhan-Hu-1-S and −0.01 kcal/mol in Omicron-S. This mutation has been analyzed through deep mutational scanning of the SARS-CoV-2 receptor binding domain. Researchers suggest that the mutation at residue F486 in Omicron BA.1/2 could lead to further evasion from antibodies found in individuals who experienced pre-Omicron and Omicron BA.1 infection. Interestingly, F486V variants have been identified among lineages of Omicron BA.4/5, contributing to a fifth epidemic wave sweeping across South Africa [26].

## 4. Discussion

### 4.1. Differences between Omicron Variants vs. Alpha, Beta, Gamma, and Delta

The Omicron RBM harbors mutations that confer increased transmissibility and infectivity of SARS-CoV-2-S compared to previous variants like alpha, beta, gamma, and delta (Table 1). The heatmaps depicting key residues in the RBM are presented in Figure 5. Most of these mutations are located in the S protein, which allows it to fit securely into the ACE2 receptor, much like a precisely crafted key into a lock [24]. This facilitates easier access for virions to host cells, resulting in greater transmissibility among people. It is hypothesized that the rapid mutation rate leading to the emergence of the Omicron variant may be due to its prolonged persistence in immunocompromised individuals, such as those with HIV/AIDS. Regions with high HIV infection rates, like Southern Africa, are suggested as potential origins for this strain [1]. Amino acid substitutions in the RBM contribute to greater escape from immune responses initiated by vaccines developed for preceding variants. Notable amino acid changes include Q498R and F486V (Table 1). As a result, Omicron has increased resistance to both vaccines and previous variants of the virus, leading to increased virulence. Unlike the alpha and delta variants, which are associated with severe illness and high fatality rates alongside high transmissibility, the Omicron variant displays lower lethality, but exhibits an exceptionally rapid transmission rate. Evolutionary analyses indicate that Omicron likely diverged neither from neither alpha, beta, gamma, nor delta variants, further highlighting its unique evolutionary trajectory [27]. As shown in Figure 5, our computational evidence predicts changes in binding affinity for specific missense mutations, such as Q498R (ΔΔΔG = −1.47 kcal/mol in Wuhan-Hu-1-S). Numerous studies indicate that the Omicron variant is significantly more infectious than its ancestral variant and other variants like beta and delta, primarily due to its extensive mutations in the RBM region [28]. Furthermore, two newly identified sub-lineages within Omicron, BA.4 and BA.5, exhibit even higher resistance to a wide range of monoclonal antibodies compared to the earlier BA.1 and BA.2 strains, suggesting an unprecedented level of infectivity for these viral mutations [29].

### 4.2. The RBM Is More Divergent Than the RBD in Omicron

Research indicates that the RBD, particularly the immunodominant RBM, exhibits high variability, enabling the virus to evade detection by the antibody response. Several studies have demonstrated that SARS-CoV-2 has a low genetic barrier to developing resistance to neutralizing antibodies targeting the RBD region [30]. This is due to the emergence of several independent mutations in the RBD region of the vesicular stomatitis virus SARS-CoV-2 chimeric system when exposed to antibody pressure. Hence, it is vital to monitor mutations in the RBD region, as they could significantly affect the progress of COVID-19 and its treatment options [31]. The RBM charge within the flexible loop region might have been altered by mutations, making it easier for binding to occur. The K417N, E484K, E484Q, and F490S, all known to aid antibody evasion, have minimal effects on ACE2 binding affinity, indicating no loss of fitness [32]. Regarding RBM-ACE2 binding affinity, interfaces between RBM and ACE2 show a significant increase in hydrogen bonds, indicating specific interactions between the two proteins. The shared residue, differing between SARS-CoV-2 and SARS-CoV, interacts with the same set of amino acids in ACE2, suggesting a comparable level of affinity for ACE2 between the two viruses. A key distinction in complex structures lies in the location of K417 in Wuhan-Hu-1-S, which, although outside the RBM, forms salt-bridge interactions with the D30 of ACE2. In contrast, the equivalent position in SARS-CoV-1 contains a valine residue, unable to form similar salt bridges. This slight difference may account for the slightly greater affinity between Wuhan-Hu-1-S RBD and ACE2 compared to SARS-CoV-1 [33]. In our analysis, the K417N mutation is observed to enhance the binding affinity (ΔΔΔG = −0.9231 kcal/mol), suggesting its potential impact on the virus’s behavior and interaction with host cells.

### 4.3. Residue Changes G431W and P507W May Cause the Highest Destabilizations in Wuhan-Hu-1-S/Omicron-S RBM and RBD

Among all of the mutations present in Omicron-S, the glycine mutation G431W seems to cause the highest increase in positive folding energy with a value of ΔΔG = 55.82 kcal/mol. The mutations in glycine residue G431 have the most significant destabilizing effects on the RBD of the S-protein (mean ΔΔG = 24.65 kcal/mol). When glycine (G), the smallest amino acid, is substituted with larger amino acids, it triggers unfavorable conformational changes, leading to protein instability [34]. Similarly, the mutation P507W seems to cause the second-highest destabilization (ΔΔG value = 52.01 kcal/mol). Proline has a unique side chain that loops back and reattaches to the parent amino group, which is different from other amino acids. Due to this structure, proline lacks a hydrogen atom on its nitrogen when it is part of a polypeptide chain. In contrast, tryptophan (W), characterized by the highest relative mutability among mutant residues, features an indole ring bound by a methylene group in its side chain. This structural configuration contributes to its substantial size and hydrophobic properties, further amplifying its destabilization effects [21]. These mutations might give the virus an advantage in evading detection by the immune system and increasing its contagiousness, likely by inducing specific structural modifications. Most of the sequence variations reported in the Omicron Variant are single nucleotide polymorphisms, which have resulted in an 85% reduction in the efficacy of neutralizing antibodies [35]. Viruses continually evolve, giving rise to new variants, some of which may possess characteristics that promote wider transmission or increased severity. However, the sheer number of infected individuals and the diverse array of potential targets raises concerns that new variants could undermine both vaccines and therapeutic interventions.

## 5. Conclusions

The S protein plays a pivotal role in viral infectivity, with mutations in its sequence present in all strains of interest or concern. Our research highlights the mutations G431W and P507W as possibly exhibiting the highest destabilizing effects in both Wuhan-Hu-1-S and Omicron-S. The emergence of various viral variants has led to increased infectivity and transmission rates, possibly driven by their enhanced affinity for the ACE2 receptor. We predict that mutations in Omicron-S display a higher tendency to enhance their binding affinity compared to Wuhan-Hu-1-S. Understanding the interaction between the RBD/RBM and ACE2 is essential not only for comprehending the behavior of different virus strains, but also for designing the effective therapeutic neutralizing antibodies. This knowledge can guide the development of the next generation of neutralizing antibodies capable of counteracting the immune-evading mechanisms of future SARS-CoV-2 variants.

## Figures and Tables

**Table 1:** New Omicron RBD mutations.

Synonyms	Lineages	Origin	RBM Mutations
Alpha	B.1.1.7	United Kingdom, October 2020	E484A, N501Y
Beta	B.1.351	E. Cape, South Africa, October 2020	K417N, E484K, N501Y
Gamma	P.1	Manaus, Brazil, March 2020	K417N/T, E484K, N501Y
Delta	B.1.617.2	Maharashtra, India, April 2020	L452R, K478T
Omicron	BA.4, BA.5	South Africa, January 2022	L452R, F486V
Omicron	BQ.1.1	United Kingdom, November 2022	D405N, R408S, K417N, N440K, K444T, G446S, L452R, N460K, S477N, T478K, E484A, F486V, Q498R, N501Y, Y505H
Omicron	XBB.1.5	United States, October 2022	D405N, R408S, K417N, N440K, K444T, V445P, G446S, N460K, S477N, T478K, E484A, F486V, F490S, Q498R, N501Y, Y505H

## Data Availability

The original contributions presented in the study are included in the article, further inquiries can be directed to the corresponding authors.
